# Macranthoside B Suppresses the Growth of Adenocarcinoma of Esophagogastric Junction by Regulating Iron Homeostasis and Ferroptosis through NRF2 Inhibition

**DOI:** 10.2174/0115680096370291250109103853

**Published:** 2025-01-22

**Authors:** Lingling Wang, Guangzhao Pan, Sichao Tian, Che Zhang, Fangfang Tao, Jiang-Jiang Qin

**Affiliations:** 1 School of Life Sciences, Tianjin University, Tianjin 300000, China;; 2 Hangzhou Institute of Medicine (HIM), Chinese Academy of Sciences, Hangzhou, Zhejiang, 310018, China;; 3 Institute of Medicinal Plant Development, Chinese Academy of Medical Sciences and Peking Union Medical College, Beijing, 100193, China;; 4 Zhejiang Key Laboratory of Blood-Stasis-Toxin Syndrome, Zhejiang Chinese Medical University, Hangzhou, 310000, China

**Keywords:** Macranthoside B, AEG, NRF2, NCOA4, ferritinophagy, iron homeostasis

## Abstract

**Background:**

Macranthoside B (MB) is a saponin compound extracted from honeysuckle that has been reported to exhibit significant medicinal values, particularly anti-tumor activities. This study aimed to evaluate the anticancer efficacy of MB in treating adenocarcinoma of the esophagogastric junction (AEG) and elucidate its underlying mechanisms.

**Methods:**

Three AEG cell lines and normal gastric epithelial cells were used to assess the anticancer activity of MB *in vitro*. A series of experiments, including RNA sequencing (RNA-seq) analysis, transmission electron microscopy (TEM), immunofluorescence, and western blot assay, were conducted to validate the molecular mechanisms by which MB may mediate these physiological changes. Finally, we used shRNA assays to silence the key gene driving these changes and examined the expression of molecules involved in the affected pathways.

**Results:**

MB exhibited significant anti-AEG cell activity with IC_50_ values ranging from 9.5 to 12.7 μM. RNA-seq results indicated that MB treatment in AEG cells significantly altered mRNA levels of autophagy- and ferroptosis-related genes. Further experiments revealed that MB treatment led to the up-regulation of lipid reactive oxygen species (Lip-ROS), oxidative stress-related pathway genes, and LC3B-labeled autophagic vesicles in AEG cells. Moreover, MB mediated NCOA4-dependent ferritinophagy, disrupting iron homeostasis and causing subsequent ferroptosis. We further confirmed that the intrinsic connection between autophagy and ferroptosis was due to the inhibition of NRF2 by MB. The inhibition of NRF2 by MB triggered transcriptional repression of its downstream effector molecules HERC2 and VAMP8, thus stabilizing NCOA4.

**Conclusion:**

This study demonstrated MB to inhibit AEG cell growth by regulating iron homeostasis and inducing ferroptosis through the inhibition of NRF2, providing a basis for the development of novel drugs for AEG treatment.

## 1 INTRODUCTION

Adenocarcinoma of the esophagogastric junction (AEG) has a unique localization and biological behavior [1]. In recent decades, the incidence of AEG has increased in Western and Asian countries, with over 1.5 million new cases each year [2, 3]. Currently, the most effective treatment for AEG includes a full range of therapies, such as surgical removal, chemotherapy, and immunotherapy [4]. However, the majority of AEG patients are diagnosed with locally advanced tumors or distant metastases, rendering them unsuitable for surgical intervention [5]. With advances in immunotherapy, immune checkpoint inhibitors have been developed, leading to notable progress in AEG immunotherapy [6]. Nevertheless, due to the diversity and complexity of the immune microenvironment, along with significant tumor heterogeneity, many drugs have failed in phase II clinical trials [7], and effective AEG treatment continues to face numerous challenges [8].

**Fig. (1):**
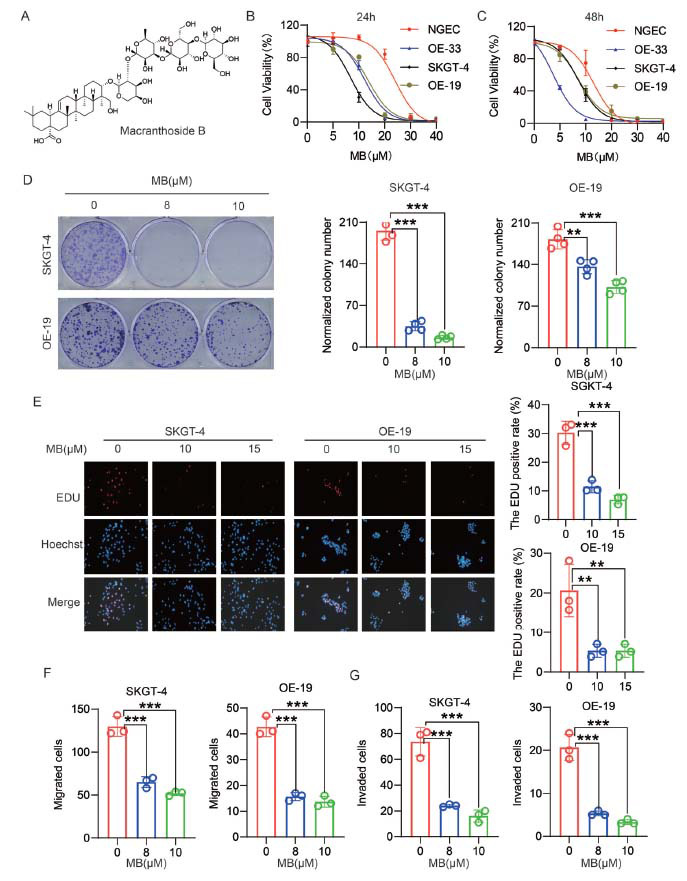
MB exhibited anti-AEG activities *in vitro*. (**A**) Chemical structure of MB. (**B-C**) The effects of MB on the viability of three AEG cell lines and a normal cell line were evaluated by 24-h and 48-h CCK-8 assays. (**D**) Colony formation assays were performed, and the number of clones in each group was examined and presented as a histogram. (**E**) The EDU staining images in SKGT-4 and OE-19 cells exposed to DMSO or MB (15 μM) for 48 h. A histogram displayed the rates of EDU positivity for each group. (**F-G**) The numbers of migrated and invaded cells in each group were depicted in a histogram. The data presented represent mean ± SD of three separate experiments. *p < 0.05, **p < 0.01, ***p < 0.001.

Macranthoside B (MB, Fig. **1A**) is a saponin compound extracted from honeysuckle with bactericidal, anti-inflammatory, and antitumor activities [9]. Previous studies have demonstrated MB’s potential as a chemotherapeutic agent for ovarian cancer, particularly targeting the ROS/AMPK/mTOR signaling pathway [10]. MB has also been shown to inhibit the growth of colorectal cancer cells by stimulating ROS-induced apoptosis [11]. Additionally, MB inhibits Hepa1-6 cell growth in a dose-dependent manner and induces oxidative stress [12]. It also exerts various suppressive effects on leukemia cells [13]. Mechanistic studies suggest that MB’s anticancer activity may result from its ability to increase ROS levels and induce oxidative stress in cells, which in turn leads to lipid peroxidation, a hallmark of ferroptosis. However, whether MB can induce ferroptosis in cancer cells is yet to be determined.

In this study, we investigated the anti-AEG activity of MB *in vitro* and its underlying anticancer mechanisms. Our findings may provide an experimental basis for clarifying the molecular mechanism by which the Traditional Chinese Medicine (TCM) honeysuckle acts against AEG and suggest a potential drug and novel strategy for AEG prevention and treatment, which holds important clinical significance.

## 2 MATERIALS AND METHODS

### 2.1 Cell Lines and Culture

The cell line 293T, the normal gastric epithelial cell line NGEC, and the AEG cell lines SKGT-4, OE-19, and OE-33 were purchased from the American Type Culture Collection (ATCC; Rockville, MD, USA). NGEC, SKGT-4, OE-19, and OE-33 were cultured in RPMI 1640 medium (Gibco/Life Technologies, Darmstadt, Germany), while 293T was cultured in DMEM medium (Gibco/Life Technologies, Darmstadt, Germany), both supplemented with 10% fetal bovine serum (FBS; Thermo Fisher Scientific, Waltham, MA, USA). Each cell culture medium was supplemented with 1% penicillin and streptomycin (Thermo Fisher Scientific, Waltham, MA, USA) and was maintained at 37°C in a humidified environment with 95% air and 5% CO_2_.

### 2.2 Reagents and Antibodies

The purity of MB was determined to be 99.8%, and it was purchased from Chengdu Biopurify Phytochemicals Ltd. (Chengdu, China). The following antibodies were obtained from Cell Signaling Technology (CST; Boston, USA): anti-GAPDH (#5174, 1:1000 dilution), anti-p62 (#5114, 1:1000 dilution), anti-NCOA4 (#66849, 1:1000 dilution), anti-FTH1 (#4393, 1:1000 dilution), and anti-NRF2 (#12721, 1:1000 dilution). Additionally, the following antibodies were also used in this study: anti-FLAG (#192890, 1:1000 dilution, Sigma-Aldrich), anti-β-actin (#0003, 1:1000 dilution, Beyotime Biotechnology), and the anti-LC3B antibody (#192890, 1:1000 dilution, Abcam).

### 2.3 Cell Viability

The CCK-8 assays were performed, as described previously [14, 15]. Briefly, 3,000 cells were seeded in each well of the 96-well plates. Cells were treated with MB (0, 5, 10, 20, 30, or 40 μM) for 24 h and 48 h. Subsequently, the CCK-8 solution (Biosharp, China) was added to detect the absorbance value, and cell viability was evaluated using GraphPad Prism 8.

### 2.4 Colony Formation Assay

The colony formation assay was conducted, as described in a previous report [16]. Briefly, SKGT-4 and OE-19 cells were cultured in 6-well plates (1000 cells/well) and treated with different concentrations of MB (0, 8, or 10 μM) for 24 h. The medium was then replaced with a fresh, drug-free medium, and the cells were allowed to grow for an additional 15-20 days until the colonies were visible to the unaided eye. Colonies were fixed with 4% paraformaldehyde (Biosharp, China) and stained with 2.5% crystal violet for visualization (Beyotime Biotechnology, China).

### 2.5 5-ethynyl-2’-deoxyuridine (EDU) Staining Assay

The EDU staining assay was performed, as described previously [17]. The cells were seeded into 24-well plates (3 × 10^4^ cells/well) and incubated overnight, followed by treatment with MB at the indicated concentrations (0, 10, or 15 μM) at 37°C for 48 h. The treated cells were then washed, fixed, and stained according to the manufacturer’s instructions. Finally, the EDU-positive cells were counted from the microscopic field of view.

### 2.6 Migration and Invasion Assays

The migration and invasion abilities of AEG cells were assessed through transwell assay [14, 18]. A total of 5 × 10^4^ cells were placed in the upper well of a Boyden chamber (Corning, United States) with or without a layer of Matrigel (BD Biosciences, USA) and treated with MB (0, 8, or 10 μM) for 36 h. The invaded and migrated cells were then stained with a 2.5% crystal violet staining solution (Solarbio, China), followed by photographing and counting.

### 2.7 RNA-sequencing

SKGT-4 cells were incubated with either 15 μM MB or DMSO for 48 h. Subsequently, the samples were collected, and total RNA was extracted from the cells using a Trizol assay. Transcriptome sequencing and RNA sample analysis were performed by Novogene (Beijing, China). Sequencing of the RNA libraries was conducted using a HiSeq system (Illumina, San Diego, CA, United States) with a 150-base pair paired-end sequencing protocol. Genes exhibiting differential expression were screened using a *p*-value threshold to ensure a false discovery rate (FDR) of 0.05 and at least a twofold variation in expression, as previously described [19].

### 2.8 Western Blot Assay

Western blot assays were carried out, as previously described [18]. The cell samples were suspended in RIPA lysis buffer (Beyotime, China), and protein concentrations were evaluated using the BCA protein assay kit (Beyotime, China). The samples were then quantified to the same concentration, and the targeted proteins were separated by SDS‐PAGE and transferred onto a polyvinylidene fluoride (PVDF) membrane (Millipore, Germany). The membranes were blocked with 5% skim milk, and then incubated with the primary antibody, followed by the secondary antibody, according to the manufacturer's instructions (#7076; #7074, CST). The ECL chemiluminescent substrate reagent kit (Biosharp, Hefei, China) was used to detect protein bands.

### 2.9 Quantitative Real-time PCR

SKGT-4 and OE-19 cells were incubated with MB at 37°C for 48 h. Total mRNA was extracted using the RNA-Quick purification kit (YiShan Biotechnology Co. Ltd., Shanghai, China) according to the manufacturer's guidelines. Quantitative real-time PCR (qRT-PCR) was performed using 2 × Super SYBR Green qPCR Master Mix (YiShan Biotechnology Co. LTD, China). Primer sequences were as follows: Human-VAMP8: 5′-AATGATCGTGTGCGGAACCT-3′ (forward) and 5′-GTGCTCAGATGTGGCTTCCA-3′ (reverse); Human-HERC2: 5′-GCCTCGACTCCAAATGGT TG-3′ (forward) and 5′-GACTCCTGCAACAGCTCACT-3′ (reverse); Human-GAPDH: 5′-CTGACTTCAACAGCGAC ACC-3′ (forward) and 5′-TGCTGTAGCCAAATTCGTT GT-3′ (reverse).

### 2.10 Lipid Peroxidation Assay

For the lipid peroxidation assay [20], SKGT-4 and OE-19 cells were cultured and exposed to different concentrations of MB or DMSO for 48 h. The cells were then collected and reconstituted in 1 mL PBS mixed with 2 µmol/L C11-BODIPY581/591 (Invitrogen, USA) and incubated at 37°C for 20 min. The cells were analyzed using flow cytometry (Thermo Fisher Scientific, Attune NxT, USA). Data analysis was performed using FlowJo.

### 2.11 Ferro Orange Assay

SKGT-4 and OE-19 cells were cultured in small laser confocal dishes, which were allowed to stabilize for 24 h. The cells were then randomly assigned to control and MB-treated groups and treated for 48 h. After three washes with PBS, a suitable amount of FerroOrange dye (DOJINDO, Japan) was added and incubated at 37°C in the dark for 20 min. A confocal laser scanning microscope (Leica Microsystems, Wetzlar, Germany) was used to examine the cells.

### 2.12 Immunofluorescence

SKGT-4 and OE-19 cells (3 × 10^4^ cells/well) were plated onto glass coverslips overnight and treated with MB (15 μM) for 48 h. The treated cells were fixed with 4% paraformaldehyde for 20 min, followed by a 15-minute incubation with 0.3% Triton X-100. The cells were then incubated with a 5% solution of goat serum, followed by incubation with the primary antibodies: LC3B (#192890, 1:1000 dilution, Abcam), anti-LAMP1 (#9091, 1:400 dilution, CST), or NRF2 (#80593, 1:500 dilution, Proteintech), and subsequently with a secondary antibody. The coverslips were mounted using an anti-fade solution containing 4’,6-diamino-2-phenylindole (DAPI), and photographs were taken using a confocal microscope (Leica Microsystems, Wetzlar, Germany).

### 2.13 Transfection and Infection

Transfection was performed using Lipofectamine^TM^ 2000 (#11668030, ThermoFisher Scientific) according to the manufacturer's protocol. Cells were transfected with plasmids encoding mRFP-eGFP-LC3B, pcDNA3.1-3xFlag-NRF2, and a vehicle plasmid, respectively. The NCOA4- and GFP-specific shRNA primers were purchased from Sangon Biotech (Shanghai, China) and integrated into the pLKO.1 vector. To prepare the lentivirus, 293T cells underwent co-transfection with the packaging plasmids pLP1, pLP2, and pLP/VSVG (Invitrogen), along with their respective shRNA plasmids (shNCOA4 1#, shNCOA4 2# and shGFP). The target sequences for the shRNA were as follows:

shNCOA4 1#: 5'-CCGGCCGGGCTGAACAGCAAATT AACTCGAGTTAATTTGCTGTTCAGCCCGGTTTTTG-3' (forward) and 5'-AATTCAAAAACCGGGCTGAACAGCA AATTAACTCGAGTTAATTTGCTGTTCAGCCCGG-3' (reverse); shNCOA4 2#: 5'-CCGGCGGGCTGAACAGCAA ATTAAACTCGAGTTTAATTTGCTGTTCAGCCCGTTTT TG-3' (forward) and 5'-AATTCAAAAACGGGCTGAACA GCAAATTAAACTCGAGTTTAATTTGCTGTTCAGCCC G-3' (reverse). shGFP: 5'-CCGGTCTAAAGGTGAAGAAT TATTCCTCGAGGAATAATTCTTCACCTTTAGATTTTT G-3' (forward), and 5'-AATTCAAAAATCTAAAGGTGAA GAATTATTCCTCGAGGAATAATTCTTCACCTTTAGA-3' (reverse). The transfection assay was carried out as previously outlined [18]. Following a 48-h transfection period, the viral supernatant was gathered to infect SKGT-4 cells. Furthermore, a concentration of 2 mg/mL of puromycin was employed in the selection of NCOA4-stable knockdown cells.

### 2.14 Statistical Analysis

Data are presented as mean ± standard deviation for each of the three duplicates. The Student’s t-test was performed using GraphPad Prism 8.0 to identify significant differences between the groups. Asterisks indicate statistically significant differences (*p < 0.05, **p < 0.01, ***p < 0.001).

## 3 RESULTS

### 3.1 MB Inhibits the Proliferation, Migration, and Invasion of AEG Cells *In Vitro*

Initially, the *in vitro* experiments were conducted to evaluate the anti-AEG activities of MB. CCK-8 assays were performed to examine the effects of MB on the viability of a normal human gastric epithelial cell line (NGEC) and three AEG cell lines (SKGT-4, OE-19, and OE-33). As shown in Fig. (**1B** and **C**), MB inhibited the viability of AEG cells in a concentration-dependent manner, while the normal NGEC cells appeared less sensitive to MB treatment. The IC_50_ values for NGEC, SKGT-4, OE-19, and OE-33 were determined to be 22.80, 9.51, 12.70, and 10.98 μM for 24 h treatment, and 11.69, 8.59, 8.47, and 4.74 μM for 48 h treatment, respectively. Considering the similar sensitivity of SKGT-4 and OE-19 to the treatment of MB, we selected these two cell lines for further studies. The EDU assay and colony formation assay were performed, and the results showed that MB inhibited proliferation in a concentration-dependent manner in SKGT-4 and OE-19 cell lines (Fig. **1D-E**). Transwell assays were also performed, and the results demonstrated that MB markedly hindered the migration and invasion of SKGT-4 and OE-19 cell lines (Fig. **1F-G**). Overall, these results suggested that MB significantly inhibited the viability, proliferation, migration, and invasion of AEG cells.

### 3.2 RNA-sequencing Analysis Indicated that MB Induced Ferroptosis and Autophagy in AEG Cells

**Fig. (2):**
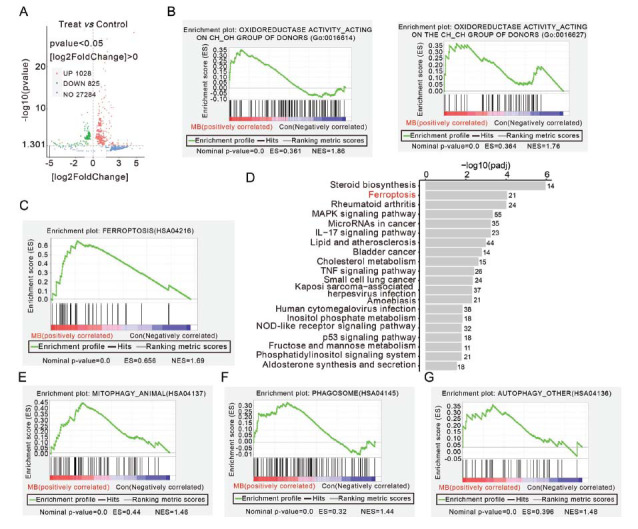
RNA-seq analysis after MB treatment in SKGT-4 cells. (**A**) The volcano plot depicted the genes with varied expression levels between MB-treated and DMSO-treated SKGT-4 cells. (**B**) The enrichment of oxidoreductase activity in MB-treated (15 μM) SKGT-4 cells *via* GSEA analysis. (**C-D**) GSEA and GO enrichment analyses were performed to analyze the ferroptosis of MB-treated and DMSO-treated cells. GSEA analysis of the autophagy-regulated processes of mitophagy (**E**), phagosome (**F**), and autophagy (**G**) in MB-treated (15 μM) and DMSO-treated cells.

To explore the potential molecular mechanisms underlying MB’s anti-AEG activity, RNA sequencing (RNA-seq) was conducted to evaluate the changes in cellular mRNA levels after MB treatment. As shown in Fig. (**2A**), the transcript levels of nearly 1800 genes were significantly altered by MB treatment. Compared to the control group, the MB group exhibited the up-regulation of 1028 genes and the down-regulation of 825 genes. Gene set enrichment analysis (GSEA) results based on different gene sets showed that MB treatment activated oxidoreductase activity and upregulated the expression of many related genes (Fig. **2B**). Furthermore, Gene ontology (GO) enrichment analysis suggested the onset of ferroptosis after MB treatment (Fig. **2C-D**). It has been reported that oxidoreductases catalyze the oxidation or reduction of substrates, and their activation indicates that the cell is in an active state of oxidation or reduction [21, 22]. This suggests that the MB-mediated activation of oxidative stress might be related to the induction of ferroptosis. The GSEA results also indicated that several regulatory genes involved in autophagy-regulated processes were significantly upregulated after MB treatment. These genes were enriched in physiological processes related to mitochondrial autophagy (Fig. **2E**), endocytosis (Fig. **2F**), and autophagy (Fig. **2G**).

### 3.3 MB Induced Ferroptosis by Mediating Cellular Lip-ROS Accumulation

**Fig. (3):**
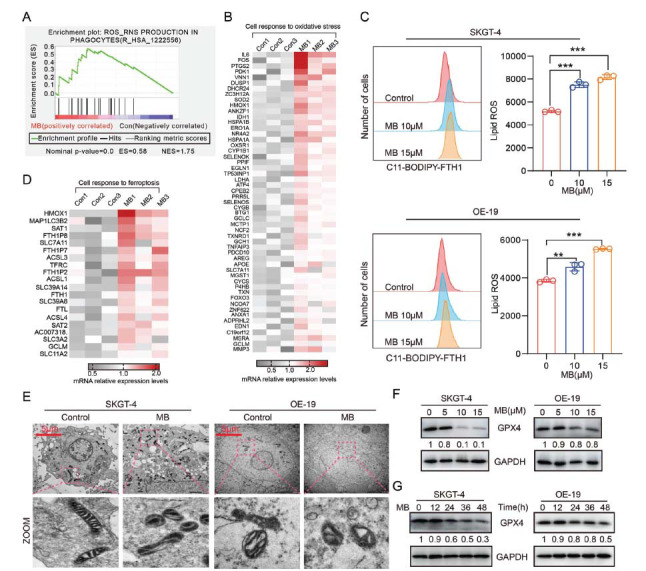
MB induced ferroptosis in AEG cells. (**A**) GSEA analysis of ROS activity in cells treated with MB or DMSO. (**B**) Relative mRNA expression levels of cell response to oxidative stress-related genes based on transcriptome data analysis. (**C**) A lipid peroxidation assay was performed on MB-treated SKGT-4 and OE-19 cells, followed by quantitative evaluation. (**D**) Relative mRNA expression levels of ferroptosis-related genes based on transcriptome data analysis. (**E**) The mitochondrial morphology was observed using a transmission electron microscope after 48 h MB treatment. (**F**) The concentration-dependent and (**G**) time-dependent inhibitory effects of MB on the protein expression of GPX4 in SKGT-4 and OE-19 cells by western blot analysis. The relative quantitation of protein expression. *p < 0.05, **p < 0.01, ***p < 0.001.

Ferroptosis occurs due to an imbalance between the consumption and production of lipid peroxides, leading to the accumulation of substantial quantities of lipid reactive oxygen species (Lip-ROS). The buildup of intracellular iron and Lip-ROS are two key factors contributing to the development of ferroptosis [23, 24]. To explore whether MB can activate ferroptosis *via* Lip-ROS accumulation, we next analyzed the ROS production after MB treatment in AEG cells. As shown in Fig. (**3A**), the GSEA results based on RNA-seq analyses indicated that the ROS-related pathways significantly changed, and the genes associated with cell response to oxidative stress were also remarkably upregulated in MB-treated cells (Fig. **3B**). Furthermore, using the C11-BODIPY, a dye employed to identify Lip-ROS in cells [25], it was clearly observed that cellular ROS levels were upregulated in a concentration-dependent manner (Fig. **3C**). Simultaneously, genes involved in regulating the cellular response to ferroptosis were activated (Fig. **3D**).

It has been shown that mitochondrial malfunction and damage lead to oxidative stress, triggering ferroptosis [26]. To investigate whether MB could cause mitochondrial damage, transmission electron microscopy (TEM) was used to examine the submicroscopic structure of MB-treated cells. As shown in Fig. (**3E**), altered mitochondrial morphology and loss of inner membrane ridges were observed in MB-treated cells, suggesting that the mitochondria were structurally disrupted by MB in SKGT-4 and OE-19 cells. Importantly, the protein level of glutathione peroxidase 4 (GPX4) was significantly reduced after MB treatment in a concentration- and time-dependent manner (Fig. **3F** and **G**). GPX4 is an enzyme that catalyzes the reduction of phospholipid peroxides, and its inactivity is a central regulator of ferroptosis [27]. These results together suggested that MB can induce ferroptosis by mediating cellular Lip-ROS accumulation.

### 3.4 MB Activated Autophagy but Impaired the Flux

**Fig. (4):**
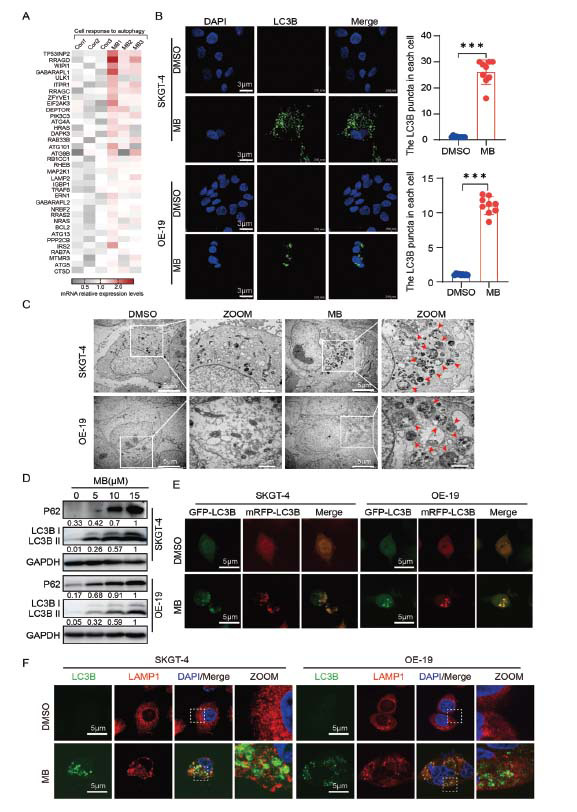
Autophagy flux was blocked by MB. (**A**) Relative mRNA expression levels of autophagy-related genes based on transcriptome data analysis. (**B**) SKGT-4 and OE-19 cells were treated with MB (15 μM) for 48 h. Following cell fixation with formalin, confocal imaging was captured. The green signal indicated the endogenous LC3B and nuclei to be counterstained with DAPI (blue). The images exemplify nine separate experiments. Calculations were made for the LC3B puncta in each cell under specified conditions. (**C**) Illustrative TEM visuals of the autophagic vacuole in AEG cells with or without MB treatment for 48 h. The red arrows indicate autophagic vacuoles. (**D**) The expression levels of LC3B and P62 in SKGT-4 and OE-19 cells after MB treatment were analyzed by western blot assays. (**E**) SKGT-4 and OE-19 cells expressing mRFP-eGFP-LC3B were treated with MB (15 μM) for 48 h, and the images were captured by confocal microscopy. (**F**) Cells were treated with MB (15 μM) for 48 h and subjected to colocalization analysis of LAMP1 and LC3B (the LAMP1 and LC3B were labeled by the LAMP1 and LC3B antibodies, respectively). The relative quantitation of protein expression. *p < 0.05, **p < 0.01, ***p < 0.001.

Previous results indicated that MB treatment might induce autophagy (Fig. **2E-G**). Further analyses of the transcriptome data revealed that many autophagy-related regulatory genes were upregulated after MB treatment in AEG cells (Fig. **4A**). Microtubule-associated proteins 1A/1B light chain 3B (LC3B) plays an irreplaceable role in the autophagy process and is commonly used as an indicator of autophagosomes [28]. As shown in Fig. (**4B**), many LC3B puncta were observed through a confocal microscope in MB-treated AEG cells, whereas few puncta were detected in the control group. Moreover, the TEM examination revealed that MB treatment caused an increase in autophagic vacuoles (autophagosomes) in AEG cells (Fig. **4C**). MB treatment also upregulated the protein levels of LC3B and sequestosome 1 (P62) (Fig. **4D**). Since P62 acts as a connector between LC3B and degradation substrates, the suppression of autophagy is associated with elevated P62 levels in mammals [29]. Consequently, these results suggest that MB likely acts as a potent inhibitor of autophagic flux.

In acidic environments, eGFP-fluorescence diminishes (such as in the autolysosome), in contrast to mRFP, which remains more stable in acidic environments [29]. The AEG cell lines were transfected with a tandem reporter construct (mRFP-eGFP-LC3B), treated with MB, and subsequently evaluated for the colocalization of GFP-LC3B and mRFP-LC3B puncta. As shown in Fig. (**4E**), exposure to MB notably led to the creation of LC3B puncta, which exhibited fluorescence intensities in both green and red, resulting in a yellow overlay. Moreover, by co-localizing LC3B (autophagosome) with lysosomal-associated membrane protein 1 (LAMP-1), a protein was located on the lysosomal membrane and commonly used as a lysosomal marker [30]. We could determine whether the autophagosomes would bind to lysosomes for assessing autophagic flux status [31]. As shown in Fig. (**4F**), we barely observed LAMP1 binding to LC3B, indicating that the autophagic flux might be impaired before the fusion of autophagosomes and lysosomes. These results further confirmed that MB activated autophagy, but prevented autophagic vesicles from binding to acidic lysosomes.

### 3.5 MB Activated NCOA4-mediated Ferritinophagy

**Fig. (5):**
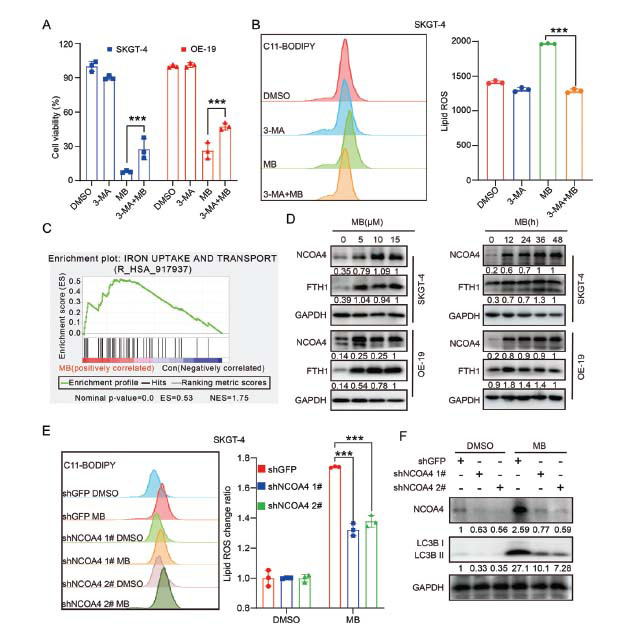
MB activated NCOA4-mediated ferritinophagy. (**A**) CCK-8 analysis of viability in SKGT-4 and OE-19 cells following treatment with MB (15 μM) in combination with or without 3-MA (10 mM) for 24 h. (**B**) Lipid peroxidation assay was performed in MB (15 μM)-treated SKGT-4 cells in combination with or without 3-MA (10 mM). (**C**) Reactome pathway analysis of the iron uptake and transport activity in MB (15 μM)-treated cells. (**D**) Concentration- and time-dependent effects of MB on the protein expression of NCOA4 and FTH1 in SKGT-4 and OE-19 cells were analyzed by western blot assays. (**E**) Lipid peroxidation assay was performed in GFP- and NCOA4-knockdown SKGT-4 cells with or without MB (15 μM) treatment. (**F**) SKGT-4 cells were transfected with GFP- or NCOA4-knockdown plasmids and further treated with MB for 24 h, which was followed by western blot analysis for the protein expression levels of LC3B and NCOA4. The relative quantitation of protein expression. *p < 0.05, **p < 0.01, ***p < 0.001.

Although the interdependence between autophagy activation and ferroptosis has been reported [32], the relationship between MB-activated autophagy and ferroptosis remains to be determined. The AEG cell lines were co-treated with MB and 3-methyladenine (3-MA), a commonly used inhibitor in the early stages of autophagy that prevents the formation of autophagosomes [29], and cell viability was subsequently evaluated. As shown in Fig. (**5A**), 3-MA significantly reduced the inhibitory effect of MB on the viability of AEG cell lines (Fig. **5A**). Meanwhile, co-treatment with 3-MA also reduced the intracellular Lip-ROS levels that were increased by MB (Fig. **5B**). These results indicated that autophagy inhibition by 3-MA reduced the inhibition of cell viability and the production of Lip-ROS by MB, suggesting that the activation of autophagy by MB contributed to MB-induced ferroptosis.

Disruption of iron homeostasis is a typical feature of ferroptosis [33]. The GSEA analysis revealed that MB treatment indeed resulted in significant alterations in the expression of genes related to iron transport and uptake (Fig. **5C**). Two key proteins that maintain cellular iron homeostasis are ferritin light chain (FTL) and ferritin heavy chain 1 (FTH1), which have iron oxidase activity and promote iron oxidation and uptake [34]. To further elucidate the link between MB-activated autophagy and ferroptosis, we examined the expression of nuclear receptor coactivator 4 (NCOA4), an adaptor protein linking autophagy activation and ferroptosis [35]. As shown in Fig. (**5D**), the levels of both NCOA4 and FTH1 proteins were upregulated in MB-treated cells, suggesting that NCOA4-mediated ferritinophagy was activated and ferritin degradation was impaired.

To clarify the association between NCOA4-mediated ferritinophagy and MB-induced ferroptosis, the AEG cell lines were transfected with an NCOA4 knockdown plasmid and then treated with MB. As expected, the Lip-ROS level in MB-treated cells was restored to some extent after the knockdown of NCOA4 (Fig. **5E**). More importantly, the increased level of LC3B induced by MB was also decreased after knocking down the expression of NCOA4 (Fig. **5F**). Collectively, these findings suggested a crucial role of NCOA4-mediated ferritinophagy in MB-induced ferroptosis.

### 3.6 MB Regulated Iron Homeostasis *via* Inhibiting NRF2

**Fig. (6):**
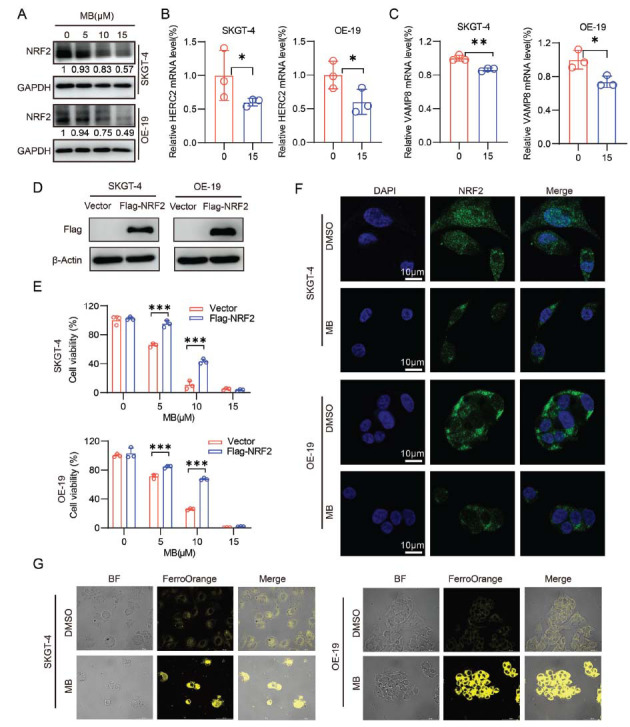
MB regulated iron homeostasis *via* inhibiting NRF2. (**A**) The effects of MB on NRF2 protein expression in SKGT-4 and OE-19 cells were analyzed by western blot assays. (**B**) SKGT-4 and OE-19 cells were exposed to MB for 48 h, and qRT-PCR analysis was performed to analyze *HERC2* mRNA levels. (**C**) SKGT-4 and OE-19 cells were exposed to MB for 48 h, and qRT-PCR analysis was performed to measure *VAMP8* mRNA levels. (**D**) Construction of NRF2 overexpression cell lines. (**E**) The effect of MB on the viability of NRF2 overexpression cell lines after 24 h treatment was detected by the CCK-8 assay. (**F**) An immunofluorescence assay was used to detect the location and content of NRF2 protein. The green signal indicates endogenous NRF2, and nuclei were counterstained with DAPI (blue). (**G**) FerroOrange assay was performed to detect Fe^2+^ content in SKGT-4 and OE-19 cells. *p < 0.05, **p < 0.01, ***p < 0.001.

As shown in Fig. (**5D**), the protein levels of FTH1 and NCOA4 were increased in MB-treated cells, indicating impaired ferritin degradation. A previous study found that a decrease in nuclear factor erythroid 2-related factor 2 (NRF2) resulted in a significant accumulation of FTH1 and NCOA4, which may provide a new insight for our study [33]. NRF2, as a key transcription factor regulating antioxidant stress, plays an important role in inducing antioxidant response, such as regulating redox balance, iron metabolism, proliferation, autophagy, proteasome degradation, DNA repair, and mitochondrial physiology [36-38]. We then examined whether the protein level of NRF2 was altered by MB treatment. Western blot analysis showed that NRF2 protein levels in MB-treated AEG cells were significantly down-regulated (Fig. **6A**). NRF2 has been reported to control iron homeostasis and ferroptosis *via* HECT and RLD domain containing E3 ubiquitin protein ligase 2 (HERC2) and vesicle-associated membrane protein 8 (VAMP8) [33]. HERC2 is an E3 ubiquitin ligase for NCOA4 [39, 40]. VAMP8 is the membrane protein that regulates the binding of autophagy and lysosome to promote autophagy [41]. We therefore examined the mRNA levels of *HERC2* and *VAMP8* in MB-treated cells. qRT-PCR analysis indicated that the mRNA levels of *HERC2* and *VAMP8* were down-regulated in SKGT-4 and OE-19 cell lines after treatment with MB (Fig. **6B** and **C**). These results suggested that the down-regulation of NRF2 by MB contributed to impaired ferritinophagy and autophagosomal accumulation of FTH1/NCOA4.

To further explore the potential functions of NRF2 in MB-treated AEG cells, we next exogenously expressed NRF2 and evaluated the sensitivity of these cells to MB treatment using cell viability assays. Our results showed that an increase in NRF2 significantly attenuated the inhibitory effect of MB on AEG cells (Fig. **6D-E**). NRF2 functions as a transcription factor with its primary site of function in the nucleus [42-44]. Therefore, we investigated whether MB could alter the nuclear localization of NRF2. We used immunofluorescence experiments to detect the content and distribution of NRF2 after MB treatment. The results showed that the NRF2 protein levels in both the cytoplasm and nucleus were significantly decreased (Fig. **6F**). Altogether, these results suggested that NRF2 may play an important role in MB-mediated cell growth inhibition and ferroptosis. Subsequently, the FerroOrange assay was performed to detect the level of Fe^2+^, and the results showed that MB significantly increased the concentration of Fe^2+^ in AEG cells (Fig. **6G**). These results indicate that MB mediated an imbalance in iron homeostasis in AEG cells.

## 4 DISCUSSION

The genesis of TCM can be traced back to extensive clinical experience guided by a related theoretical framework. This has a clear impact on the treatment of diseases and serves as an important resource for contemporary drug research and development. In this study, we identified a hederagenin saponin MB, derived from the flower bud of *Lonicera macranthoides*, which exhibited significant anti-AEG activity *in vitro* and mediated ferritinophagy and iron homeostasis *via* NRF2.

Ferroptosis is a specific type of programmed cell death and results from the peroxidation of lipids dependent on iron [45-47]. Excessive active free iron can accelerate ROS generation *via* the Fenton reaction, resulting in oxidative stress [48-50]. Clinical research has demonstrated the significant influence of ferroptosis in the initiation and development of various gastrointestinal malignancies [51-53]. Therefore, targeting ferroptosis is considered an effective strategy for the treatment of gastrointestinal malignancies. In the present study, we found that MB increased the Lip-ROS and mitochondria ferroptosis-like morphological changes in SKGT-4 and OE-19 cells, indicating ferroptosis to play an important role in MB-induced cell death.

Ferroptosis is closely related to iron metabolism [54-56]. Ferritin is composed of FTL and FTH1, which are the major sites of intracellular iron storage [56, 57]. Numerous studies have indicated the role of autophagy in regulating ferroptosis [35, 58, 59]. Our results indicated that MB triggered the activation of autophagy. NCOA4 is required for ferritinophagy, which can deliver ferritin to the lysosome and release iron ions [60]. NCOA4 controls ferritinophagy flux [61-63]. Under normal circumstances, ferritinophagy remains at low levels, but excess ferritinophagy increases cellular free iron and promotes ferroptosis [59, 64, 65]. In the process of overactivation of ferritinophagy, NCOA4 is upregulated and FTH1 is downregulated. However, in the present study, both FTH1 and NCOA4 were upregulated. We found that MB significantly decreased the protein level of NRF2, responsible for regulating homeostasis through the synthesis/degradation of ferritin, thus increasing intracellular Lip-ROS level and inducing ferroptosis. Inhibition of NRF2 by MB led to the overproduction of ferritin and the accumulation of apoferritin-NCOA4 complexes in autophagosomes, disrupting ferritin metabolism, thereby resulting in large amounts of toxic free iron accumulation and making cells more vulnerable to ferroptotic cell death [33].

Ferritinophagy is becoming increasingly important in ferroptosis and cancer, and more and more studies have shown that ferritinophagy can affect tumor growth [66-68]. In previous studies, cancer treatment targeting ferroptosis has mainly been limited to solute carrier family 7a member 11 (SLC7A11) and GPX4, and currently, therapies targeting ferritinophagy receive increasing attention [66, 69, 70]. Recent studies have shown that tetrandrine citrate can inhibit breast cancer by activating NCOA4 [71]. DpdtC, a novel iron chelator, has also been shown to promote ferritinophagy in liver and gastric cancer cells [72, 73]. These studies have demonstrated significant promise for activating ferritinophagy to treat cancer. However, medications aimed at the ferroptosis pathway remain in the preclinical stage. In the future, we anticipate an increase in medications targeting ferritinophagy. In this study, we have shown 3-MA, an inhibitor of autophagy, to reduce Lip-ROS and cell death caused by MB treatment, while knockdown of NCOA4 showed comparable results to the effects of 3-MA. These results highlight the role of ferritinophagy in MB-induced ferroptosis in AEG cells.

**Fig. (7):**
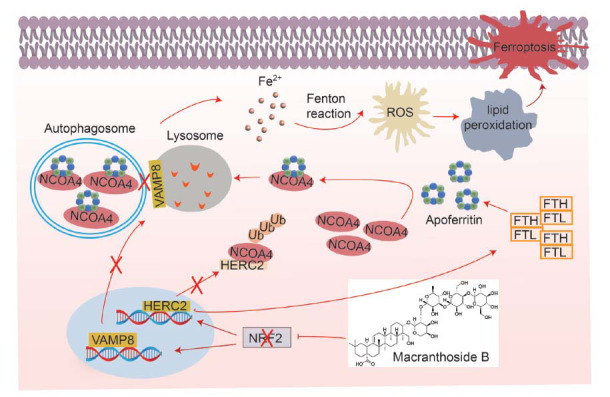
Scheme illustrating a proposed molecular mechanism by which MB triggers ferroptosis.

Importantly, due to the inhibition of NRF2 by MB, the downregulation of NRF2-dependent expression of HERC2 resulted in a sustained increase in NCOA4 level and the recruitment of apoferritin to the autophagosome. This decoupling of NCOA4 degradation from Lip-ROS, combined with the blocked autophagy flux (low VAMP8), resulted in the isolation of ferritin from unstable iron in the cytoplasm. As a consequence, an increase in Lip-ROS was observed, leading to cancer cell death (Fig. **7**).

NRF2 protein levels are modulated through several pathways. For example, NRF2 degradation is facilitated by the KEAP1-mediated ubiquitin-proteasome pathway [74]. In addition, miRNA-28 can target *NRF2* mRNA, repressing its translation and consequently reducing NRF2 protein levels [75]. The glycogen synthase kinase 3β (GSK-3β) pathway also plays a crucial role in NRF2 stability, where GSK-3β can directly phosphorylate NRF2, promoting its proteasomal degradation or inhibiting its transcriptional activity [76-78]. Our study demonstrated that MB modulated iron homeostasis by inhibiting NRF2, thereby inducing ferroptosis in AEG cells. Considering that GSK-3β can modulate ferroptosis sensitivity by regulating iron homeostasis [79], we propose further investigation into whether MB downregulates NRF2 protein levels *via* the GSK-3β signaling pathway. There were several limitations in the present study. An important limitation was the lack of *in vivo* validation. Although our *in vitro* results have been promising, it is crucial to perform animal studies to confirm the therapeutic potential and safety of MB in a living organism. In addition, this study lacked an explanation of the deeper mechanism of action of NRF2. Nevertheless, these limitations and the known findings could provide new insights for future research.

## CONCLUSION

In summary, this study demonstrated that MB inhibited AEG cell growth by regulating iron homeostasis and inducing ferroptosis through the suppression of NRF2, providing preclinical evidence for developing novel medications for AEG treatment.

## Data Availability

The data and supportive information are available within the article.

## LIST OF ABBREVIATIONS

3-MA: 3-methyladenine
AEG: Adenocarcinoma of the esophagogastric junction
DAPI: 4’, 6-diamino-2-phenylindole
EDU: 5-ethynyl-2’-deoxyuridine
FBS: Fetal bovine serum
FDR: False discovery rate
FTH1: Ferritin heavy chain 1
FTL: Ferritin light chain
GESA: Gene set enrichment analysis
GO: Gene ontology
GPX4: Glutathione peroxidase 4
GSK-3β: Glycogen synthase kinase 3β
HERC2: HECT and RLD domain containing E3 ubiquitin protein ligase 2
LAMP-1: Lysosomal-associated membrane protein 1
LC3B: Microtubule-associated proteins 1A/1B light chain 3B
Lip-ROS: Lipid reactive oxygen species
MB: Macranthoside B
NCOA4: Nuclear receptor coactivator 4
NRF2: Nuclear factor erythroid 2-related factor 2
P62: Sequestosome 1
PVDF: Polyvinylidene fluoride
qRT-PCR: Quantitative real-time PCR
RNA-seq: RNA sequencing
SLC7A11: Solute carrier family 7a member 11
TCM: Traditional Chinese medicine
TEM: Transmission electron microscope
VAMP8: Vesicle-associated membrane protein 8

## References

[1] Takeuchi H., Kitagawa Y. (2013). Adenocarcinoma of the esophagogastric junction: Territory of the esophagus or stomach, or an independent region?. Ann. Surg. Oncol..

[2] Kusano C., Gotoda T., Khor C.J., Katai H., Kato H., Taniguchi H., Shimoda T. (2008). Changing trends in the proportion of adenocarcinoma of the esophagogastric junction in a large tertiary referral center in Japan.. J. Gastroenterol. Hepatol..

[3] Liu K., Yang K., Zhang W., Chen X., Chen X., Zhang B., Chen Z., Chen J., Zhao Y., Zhou Z., Chen L., Hu J. (2016). Changes of esophagogastric junctional adenocarcinoma and gastroesophageal reflux disease among surgical patients during 1988-2012: A single-institution, high-volume experience in china.. Ann. Surg..

[4] Li S., Yuan L., Xu Z.Y., Xu J.L., Chen G.P., Guan X., Pan G.Z., Hu C., Dong J., Du Y.A., Yang L.T., Ni M.W., Jiang R.B., Zhu X., Lv H., Xu H.D., Zhang S.J., Qin J.J., Cheng X.D. (2023). Integrative proteomic characterization of adenocarcinoma of esophagogastric junction.. Nat. Commun..

[5] Ajani J.A., D’Amico T.A., Bentrem D.J., Chao J., Corvera C., Das P., Denlinger C.S., Enzinger P.C., Fanta P., Farjah F., Gerdes H., Gibson M., Glasgow R.E., Hayman J.A., Hochwald S., Hofstetter W.L., Ilson D.H., Jaroszewski D., Johung K.L., Keswani R.N., Kleinberg L.R., Leong S., Ly Q.P., Matkowskyj K.A., McNamara M., Mulcahy M.F., Paluri R.K., Park H., Perry K.A., Pimiento J., Poultsides G.A., Roses R., Strong V.E., Wiesner G., Willett C.G., Wright C.D., McMillian N.R., Pluchino L.A. (2019). Esophageal and esophagogastric junction cancers, version 2.2019, nccn clinical practice guidelines in oncology.. J. Natl. Compr. Canc. Netw..

[6] Moehler M., Högner A., Wagner A.D., Obermannova R., Alsina M., Thuss-Patience P., van Laarhoven H., Smyth E. (2022). Recent progress and current challenges of immunotherapy in advanced/metastatic esophagogastric adenocarcinoma.. Eur. J. Cancer.

[7] Cao F., Hu C., Xu Z.Y., Zhang Y.Q., Huang L., Chen J.H., Qin J.J., Cheng X.D. (2022). Current treatments and outlook in adenocarcinoma of the esophagogastric junction: A narrative review.. Ann. Transl. Med..

[8] Janjigian Y.Y., Shitara K., Moehler M., Garrido M., Salman P., Shen L., Wyrwicz L., Yamaguchi K., Skoczylas T., Campos Bragagnoli A., Liu T., Schenker M., Yanez P., Tehfe M., Kowalyszyn R., Karamouzis M.V., Bruges R., Zander T., Pazo-Cid R., Hitre E., Feeney K., Cleary J.M., Poulart V., Cullen D., Lei M., Xiao H., Kondo K., Li M., Ajani J.A. (2021). First-line nivolumab plus chemotherapy versus chemotherapy alone for advanced gastric, gastro-oesophageal junction, and oesophageal adenocarcinoma (CheckMate 649): A randomised, open-label, phase 3 trial.. Lancet.

[9] Tang X., Liu X., Zhong J., Fang R. (2021). Potential application of lonicera japonica extracts in animal production: From the perspective of intestinal health.. Front. Microbiol..

[10] Shan Y., Guan F., Zhao X., Wang M., Chen Y., Wang Q., Feng X. (2016). Macranthoside b induces apoptosis and autophagy via reactive oxygen species accumulation in human ovarian cancer a2780 cells.. Nutr. Cancer.

[11] Fan X., Rao J., Zhang Z., Li D., Cui W., Zhang J., Wang H., Tou F., Zheng Z., Shen Q. (2018). Macranthoidin b modulates key metabolic pathways to enhance ros generation and induce cytotoxicity and apoptosis in colorectal cancer.. Cell. Physiol. Biochem..

[12] Tan S., Liu Q., Yang J., Cai J., Yu M., Ji Y. (2023). Macranthoidin b (mb) promotes oxidative stress-induced inhibiting of hepa1-6 cell proliferation via selenoprotein.. Biol. Trace Elem. Res..

[13] Guan F., Shan Y., Zhao X., Zhang D., Wang M., Peng F., Xia B., Feng X. (2011). Apoptosis and membrane permeabilisation induced by macranthoside B on HL-60 cells.. Nat. Prod. Res..

[14] Wang W., Qin J.J., Voruganti S., Nijampatnam B., Velu S.E., Ruan K.H., Hu M., Zhou J., Zhang R. (2018). Discovery and characterization of dual inhibitors of mdm2 and nfat1 for pancreatic cancer therapy.. Cancer Res..

[15] Ma Y., Peng Y., Cheng S., Jin L. (2024). Targeting mgst1 makes non-small cell lung cancer cells sensitive to radiotherapy by epigenetically enhancing alox15-mediated ferroptosis.. Curr. Cancer Drug Targets.

[16] Wang W., Qin J.J., Voruganti S., Wang M.H., Sharma H., Patil S., Zhou J., Wang H., Mukhopadhyay D., Buolamwini J.K., Zhang R. (2014). Identification of a new class of MDM2 inhibitor that inhibits growth of orthotopic pancreatic tumors in mice.. Gastroenterology.

[17] Pan G., Zhang K., Geng S., Lan C., Hu X., Li C., Ji H., Li C., Hu X., Wang Y., Lv M., Cui H. (2022). PHF14 knockdown causes apoptosis by inducing DNA damage and impairing the activity of the damage response complex in colorectal cancer.. Cancer Lett..

[18] Qi S., Guan X., Zhang J., Yu D., Yu X., Li Q., Yin W., Cheng X.D., Zhang W., Qin J.J. (2022). Targeting E2 ubiquitin-conjugating enzyme UbcH5c by small molecule inhibitor suppresses pancreatic cancer growth and metastasis.. Mol. Cancer.

[19] Zhang K., Fu G., Pan G., Li C., Shen L., Hu R., Zhu S., Chen Y., Cui H. (2018). Demethylzeylasteral inhibits glioma growth by regulating the miR-30e-5p/MYBL2 axis.. Cell Death Dis..

[20] Guan X., Wang Y., Yu W., Wei Y., Lu Y., Dai E., Dong X., Zhao B., Hu C., Yuan L., Luan X., Miao K., Chen B., Cheng X.D., Zhang W., Qin J.J. (2024). Blocking ubiquitin-specific protease 7 induces ferroptosis in gastric cancer via targeting stearoyl-coa desaturase.. Adv. Sci. (Weinh.).

[21] Liu N., Xu H., Sun Q., Yu X., Chen W., Wei H., Jiang J., Xu Y., Lu W. (2021). The role of oxidative stress in hyperuricemia and xanthine oxidoreductase (xor) inhibitors.. Oxid. Med. Cell. Longev..

[22] Lei G., Zhuang L., Gan B. (2022). Targeting ferroptosis as a vulnerability in cancer.. Nat. Rev. Cancer.

[23] Rochette L., Dogon G., Rigal E., Zeller M., Cottin Y., Vergely C. (2022). Lipid peroxidation and iron metabolism: Two corner stones in the homeostasis control of ferroptosis.. Int. J. Mol. Sci..

[24] Wang Y., Liao S., Pan Z., Jiang S., Fan J., Yu S., Xue L., Yang J., Ma S., Liu T., Zhang J., Chen Y. (2022). Hydrogen sulfide alleviates particulate matter-induced emphysema and airway inflammation by suppressing ferroptosis.. Free Radic. Biol. Med..

[25] Wang X., Shen T., Lian J., Deng K., Qu C., Li E., Li G., Ren Y., Wang Z., Jiang Z., Sun X., Li X. (2023). Resveratrol reduces ROS-induced ferroptosis by activating SIRT3 and compensating the GSH/GPX4 pathway.. Mol. Med..

[26] Li J., Jia Y., Ding Y., Bai J., Cao F., Li F. (2023). The crosstalk between ferroptosis and mitochondrial dynamic regulatory networks.. Int. J. Biol. Sci..

[27] Chen P., Wu Q., Feng J., Yan L., Sun Y., Liu S., Xiang Y., Zhang M., Pan T., Chen X., Duan T., Zhai L., Zhai B., Wang W., Zhang R., Chen B., Han X., Li Y., Chen L., Liu Y., Huang X., Jin T., Zhang W., Luo H., Chen X., Li Y., Li Q., Li G., Zhang Q., Zhuo L., Yang Z., Tang H., Xie T., Ouyang X., Sui X. (2020). Erianin, a novel dibenzyl compound in Dendrobium extract, inhibits lung cancer cell growth and migration via calcium/calmodulin-dependent ferroptosis.. Signal Transduct. Target. Ther..

[28] Klionsky D.J., Abdelmohsen K., Abe A., Abedin M.J., Abeliovich H., Acevedo Arozena A. (2016). Guidelines for the use and interpretation of assays for monitoring autophagy. (3rd edition). Autophagy,.

[29] Zhou J., Li G., Zheng Y., Shen H.M., Hu X., Ming Q.L., Huang C., Li P., Gao N. (2015). A novel autophagy/mitophagy inhibitor liensinine sensitizes breast cancer cells to chemotherapy through DNM1L-mediated mitochondrial fission.. Autophagy.

[30] Cheng X.T., Xie Y.X., Zhou B., Huang N., Farfel-Becker T., Sheng Z.H. (2018). Revisiting LAMP1 as a marker for degradative autophagy-lysosomal organelles in the nervous system.. Autophagy.

[31] Yang Y., Wang Q., Song D., Zen R., Zhang L., Wang Y., Yang H., Zhang D., Jia J., Zhang J., Wang J. (2020). Lysosomal dysfunction and autophagy blockade contribute to autophagy-related cancer suppressing peptide-induced cytotoxic death of cervical cancer cells through the AMPK/mTOR pathway.. J. Exp. Clin. Cancer Res..

[32] Liu J., Kuang F., Kroemer G., Klionsky D.J., Kang R., Tang D. (2020). Autophagy-dependent ferroptosis: Machinery and regulation.. Cell Chem. Biol..

[33] Anandhan A., Dodson M., Shakya A., Chen J., Liu P., Wei Y., Tan H., Wang Q., Jiang Z., Yang K., Garcia J.G.N., Chambers S.K., Chapman E., Ooi A., Yang-Hartwich Y., Stockwell B.R., Zhang D.D. (2023). NRF2 controls iron homeostasis and ferroptosis through HERC2 and VAMP8.. Sci. Adv..

[34] Mi Y., Wei C., Sun L., Liu H., Zhang J., Luo J., Yu X., He J., Ge H., Liu P. (2023). Melatonin inhibits ferroptosis and delays age-related cataract by regulating SIRT6/p-Nrf2/GPX4 and SIRT6/NCOA4/FTH1 pathways.. Biomed. Pharmacother..

[35] Zhou B., Liu J., Kang R., Klionsky D.J., Kroemer G., Tang D. (2020). Ferroptosis is a type of autophagy-dependent cell death.. Semin. Cancer Biol..

[36] Hayes J.D., Dinkova-Kostova A.T., Tew K.D. (2020). Oxidative stress in cancer.. Cancer Cell.

[37] Rojo de la Vega M., Chapman E., Zhang D.D. (2018). Nrf2 and the hallmarks of cancer.. Cancer Cell.

[38] Tao S., Liu P., Luo G., Rojo de la Vega M., Chen H., Wu T., Tillotson J., Chapman E., Zhang D.D. (2017). P97 negatively regulates nrf2 by extracting ubiquitylated nrf2 from the keap1-cul3 e3 complex.. Mol. Cell. Biol..

[39] Moroishi T., Yamauchi T., Nishiyama M., Nakayama K.I. (2014). HERC2 targets the iron regulator FBXL5 for degradation and modulates iron metabolism.. J. Biol. Chem..

[40] Vashisht A.A., Zumbrennen K.B., Huang X., Powers D.N., Durazo A., Sun D., Bhaskaran N., Persson A., Uhlen M., Sangfelt O., Spruck C., Leibold E.A., Wohlschlegel J.A. (2009). Control of iron homeostasis by an iron-regulated ubiquitin ligase.. Science.

[41] Wang L., Diao J. (2022). VAMP8 phosphorylation regulates lysosome dynamics during autophagy.. Autophagy Rep..

[42] Tonelli C., Chio I.I.C., Tuveson D.A. (2018). Transcriptional regulation by nrf2.. Antioxid. Redox Signal..

[43] Nishizawa H., Yamanaka M., Igarashi K. (2023). Ferroptosis: regulation by competition between NRF2 and BACH1 and propagation of the death signal.. FEBS J..

[44] Modi R., McKee N., Zhang N., Alwali A., Nelson S., Lohar A., Ostafe R., Zhang D.D., Parkinson E.I. (2023). Stapled peptides as direct inhibitors of nrf2-smaf transcription factors.. J. Med. Chem..

[45] Dixon S.J., Lemberg K.M., Lamprecht M.R., Skouta R., Zaitsev E.M., Gleason C.E., Patel D.N., Bauer A.J., Cantley A.M., Yang W.S., Morrison B., Stockwell B.R. (2012). Ferroptosis: An iron-dependent form of nonapoptotic cell death.. Cell.

[46] Deng L., He S., Guo N., Tian W., Zhang W., Luo L. (2023). Molecular mechanisms of ferroptosis and relevance to inflammation.. Inflamm. Res..

[47] Yao T., Li L. (2023). The influence of microbiota on ferroptosis in intestinal diseases.. Gut Microbes.

[48] Zhou R.P., Chen Y., Wei X., Yu B., Xiong Z.G., Lu C., Hu W. (2020). Novel insights into ferroptosis: Implications for age-related diseases.. Theranostics.

[49] Jomova K., Valko M. (2011). Advances in metal-induced oxidative stress and human disease.. Toxicology.

[50] Liu J., Kang R., Tang D. (2022). Signaling pathways and defense mechanisms of ferroptosis.. FEBS J..

[51] Jiang X., Yan Q., Xie L., Xu S., Jiang K., Huang J., Wen Y., Yan Y., Zheng J., Tang S., Nie K., Zheng Z., Pan J., Liu P., Huang Y., Yan X., Zou Y., Chen X., Liu F., Li P., Zhuang K. (2021). Construction and validation of a ferroptosis-related prognostic model for gastric cancer.. J. Oncol..

[52] Liu G., Ma J., Hu G., Jin H. (2021). Identification and validation of a novel ferroptosis-related gene model for predicting the prognosis of gastric cancer patients.. PLoS One.

[53] Palzer J., Eckstein L., Slabu I., Reisen O., Neumann U.P., Roeth A.A. (2021). Iron oxide nanoparticle-based hyperthermia as a treatment option in various gastrointestinal malignancies.. Nanomaterials (Basel).

[54] Le J., Pan G., Zhang C., Chen Y., Tiwari A.K., Qin J.J. (2024). Targeting ferroptosis in gastric cancer: Strategies and opportunities.. Immunol. Rev..

[55] Hassannia B., Vandenabeele P., Vanden Berghe T. (2019). Targeting ferroptosis to iron out cancer.. Cancer Cell.

[56] Bayır H., Dixon S.J., Tyurina Y.Y., Kellum J.A., Kagan V.E. (2023). Ferroptotic mechanisms and therapeutic targeting of iron metabolism and lipid peroxidation in the kidney.. Nat. Rev. Nephrol..

[57] Qin X., Zhang J., Wang B., Xu G., Yang X., Zou Z., Yu C. (2021). Ferritinophagy is involved in the zinc oxide nanoparticles-induced ferroptosis of vascular endothelial cells.. Autophagy.

[58] Friedmann Angeli J.P., Schneider M., Proneth B., Tyurina Y.Y., Tyurin V.A., Hammond V.J., Herbach N., Aichler M., Walch A., Eggenhofer E., Basavarajappa D., Rådmark O., Kobayashi S., Seibt T., Beck H., Neff F., Esposito I., Wanke R., Förster H., Yefremova O., Heinrichmeyer M., Bornkamm G.W., Geissler E.K., Thomas S.B., Stockwell B.R., O’Donnell V.B., Kagan V.E., Schick J.A., Conrad M. (2014). Inactivation of the ferroptosis regulator Gpx4 triggers acute renal failure in mice.. Nat. Cell Biol..

[59] Hou W., Xie Y., Song X., Sun X., Lotze M.T., Zeh H.J., Kang R., Tang D. (2016). Autophagy promotes ferroptosis by degradation of ferritin.. Autophagy.

[60] Mancias J.D., Wang X., Gygi S.P., Harper J.W., Kimmelman A.C. (2014). Quantitative proteomics identifies NCOA4 as the cargo receptor mediating ferritinophagy.. Nature.

[61] Zhao H., Lu Y., Zhang J., Sun Z., Cheng C., Liu Y., Wu L., Zhang M., He W., Hao S., Li K. (2024). NCOA4 requires a [3Fe-4S] to sense and maintain the iron homeostasis.. J. Biol. Chem..

[62] Jiang J., Zhou X., Chen H., Wang X., Ruan Y., Liu X., Ma J. (2024). 18β-Glycyrrhetinic acid protects against deoxynivalenol-induced liver injury via modulating ferritinophagy and mitochondrial quality control.. J. Hazard. Mater..

[63] Guggisberg C.A., Kim J., Lee J., Chen X., Ryu M.S. (2022). Ncoa4 regulates iron recycling and responds to hepcidin activity and lipopolysaccharide in macrophages.. Antioxidants (Basel).

[64] Gryzik M., Asperti M., Denardo A., Arosio P., Poli M. (2021). NCOA4-mediated ferritinophagy promotes ferroptosis induced by erastin, but not by RSL3 in HeLa cells.. Biochim. Biophys. Acta Mol. Cell Res..

[65] Lin P.L., Tang H.H., Wu S.Y., Shaw N.S., Su C.L. (2020). Saponin formosanin c-induced ferritinophagy and ferroptosis in human hepatocellular carcinoma cells.. Antioxidants (Basel).

[66] Sun K., Li C., Liao S., Yao X., Ouyang Y., Liu Y., Wang Z., Li Z., Yao F. (2022). Ferritinophagy, a form of autophagic ferroptosis: New insights into cancer treatment.. Front. Pharmacol..

[67] Su Y., Zhao B., Zhou L., Zhang Z., Shen Y., Lv H., AlQudsy L.H.H., Shang P. (2020). Ferroptosis, a novel pharmacological mechanism of anti-cancer drugs.. Cancer Lett..

[68] Chen X., Li J., Kang R., Klionsky D.J., Tang D. (2021). Ferroptosis: Machinery and regulation.. Autophagy.

[69] Wang J., Wu N., Peng M., Oyang L., Jiang X., Peng Q., Zhou Y., He Z., Liao Q. (2023). Ferritinophagy: Research advance and clinical significance in cancers.. Cell Death Discov..

[70] Jin X., Jiang C., Zou Z., Huang H., Li X., Xu S., Tan R. (2023). Ferritinophagy in the etiopathogenic mechanism of related diseases.. J. Nutr. Biochem..

[71] Yin J., Lin Y., Fang W., Zhang X., Wei J., Hu G., Liu P., Niu J., Guo J., Zhen Y., Li J. (2022). Tetrandrine citrate suppresses breast cancer via depletion of glutathione peroxidase 4 and activation of nuclear receptor coactivator 4-mediated ferritinophagy.. Front. Pharmacol..

[72] Grignano E., Birsen R., Chapuis N., Bouscary D. (2020). From iron chelation to overload as a therapeutic strategy to induce ferroptosis in leukemic cells.. Front. Oncol..

[73] Gonciarz R.L., Collisson E.A., Renslo A.R. (2021). Ferrous iron-dependent pharmacology.. Trends Pharmacol. Sci..

[74] Baird L., Yamamoto M. (2020). The molecular mechanisms regulating the keap1-nrf2 pathway.. Mol. Cell. Biol..

[75] Yue C.F., Li L.S., Ai L., Deng J.K., Guo Y.M. (2021). sMicroRNA-28-5p acts as a metastasis suppressor in gastric cancer by targeting Nrf2.. Exp. Cell Res..

[76] Lv H., Liu Q., Wen Z., Feng H., Deng X., Ci X. (2017). Xanthohumol ameliorates lipopolysaccharide (LPS)-induced acute lung injury via induction of AMPK/GSK3β-Nrf2 signal axis.. Redox Biol..

[77] Duan J., Cui J., Yang Z., Guo C., Cao J., Xi M., Weng Y., Yin Y., Wang Y., Wei G., Qiao B., Wen A. (2019). Neuroprotective effect of Apelin 13 on ischemic stroke by activating AMPK/GSK-3β/Nrf2 signaling.. J. Neuroinflammation.

[78] Duan J., Guan Y., Mu F., Guo C., Zhang E., Yin Y., Wei G., Zhu Y., Cui J., Cao J., Weng Y., Wang Y., Xi M., Wen A. (2017). Protective effect of butin against ischemia/reperfusion-induced myocardial injury in diabetic mice: Involvement of the AMPK/GSK-3β/Nrf2 signaling pathway.. Sci. Rep..

[79] Wang L., Ouyang S., Li B., Wu H., Wang F. (2021). GSK-3β manipulates ferroptosis sensitivity by dominating iron homeostasis.. Cell Death Discov..

